# Anti–early antigen Epstein-Barr virus titer and atherosclerosis in relation to vascular endothelial growth factor (VEGF) polymorphism rs3025039 among older Japanese individuals

**DOI:** 10.1265/ehpm.25-00334

**Published:** 2025-10-28

**Authors:** Yuji Shimizu, Hirotomo Yamanashi, Shin-Ya Kawashiri, Yuko Noguchi, Nagisa Sasaki, Seiko Nakamichi, Kazuhiko Arima, Yasuhiro Nagata, Takahiro Maeda

**Affiliations:** 1 Epidemiology Section, Division of Public Health, Osaka Institute of Public Health, Osaka, Japan; 2 Department of General Medicine, Nagasaki University Graduate School of Biomedical Sciences, Nagasaki, Japan; 3 Leading Medical Research Core Unit, Nagasaki University Graduate School of Biomedical Sciences, Nagasaki, Japan; 4 Department of Community Medicine, Nagasaki University Graduate School of Biomedical Sciences, Nagasaki, Japan; 5 Nagasaki University Health Center, Nagasaki, Japan; 6 Department of Public Health, Nagasaki University Graduate School of Biomedical Sciences, Nagasaki, Japan; 7 Department of Island and Community Medicine, Nagasaki University Graduate School of Biomedical Sciences, Nagasaki, Japan

**Keywords:** Angiogenesis, Atherosclerosis, VEGF, EB virus, SNP, rs3025039

## Abstract

**Background:**

Epstein-Barr (EB) virus infection stimulates the production of vascular endothelial growth factor (VEGF), which contributes to the progression of angiogenesis. Angiogenesis plays an important role in the development of atherosclerosis. Since serum anti-early antigen EB virus IgG (EBV EA-IgG) titer is a sign of active EB virus infection, EBV EA-IgG titer could be associated with atherosclerosis. The number of minor (T) alleles in VEGF polymorphism rs3025039 has been reported to be inversely associated with serum VEGF concentration, suggesting that rs3025039 might have a strong influence on the association between EBV EA-IgG titer and atherosclerosis. By focusing on the role of VEGF in the development of atherosclerosis, this study aimed to investigate the association between active EB virus infection and atherosclerosis.

**Methods:**

A cross-sectional study of 2,661 older Japanese individuals aged 60–89 years who participated in annual health check-ups during 2017–2019 was conducted. Logistic regression was used to evaluate the association between EBV EA-IgG titer and atherosclerosis in relation to rs3025039 genotype. The influence of rs3025039 (T) allele carrier status on the association between EBV EA-IgG titer and atherosclerosis was also evaluated by using logistic regression.

**Results:**

Among rs3025039 CC-homozygotes, with the lowest EBV EA-IgG titer tertile as the reference, the multivariable odds ratio (95% confidence interval) was 1.11 (0.82, 1.50) for the medium tertile and 1.07 (0.78, 1.47) for the high tertile. Among rs3025039 (T) allele carriers, the corresponding values were 1.44 (0.88, 2.36) and 1.88 (1.15, 3.05), respectively. There was a significant interaction between rs3025039 (T) allele carrier status and the association between EBV EA-IgG titer and atherosclerosis (adjusted p = 0.0497).

**Conclusion:**

EBV EA-IgG titer was significantly positively associated with atherosclerosis only among participants who are genetically less likely to have progressive angiogenesis. An angiogenesis-related genetic factor was revealed as a determinant of the association between EBV EA-IgG titer and atherosclerosis. These findings introduce a novel concept that could explain the association between viral infection and atherosclerosis.

## 1. Introduction

Epstein-Barr (EB) virus is found around the world. Approximately 95% of the world’s population are infected with EB virus [1]. EB virus is a known cause of serious disease, including a wide spectrum of malignancies such as nasopharyngeal carcinoma, gastric carcinoma, Hodgkin lymphoma, Burkitt lymphoma, and diffuse large B-cell lymphoma [2]; systemic autoimmune disease [3]; and chronic active EB virus disease [4]. However, most individuals have inactive latent infection.

Cells infected with EB virus upregulate vascular endothelial growth factor (VEGF) [5–7], which stimulates angiogenesis [8]. Angiogenesis is an important process in the development of atherosclerosis [9, 10]. Therefore, EB virus infection could be associated with atherosclerosis.

Since higher serum anti-early antigen EB virus IgG (EBV EA-IgG) titer is a sign of active EB virus infection, EBV EA-IgG titer, by reflecting VEGF production, could be positively associated with atherosclerosis.

Aging is a process that increases hypoxia and oxidative stress [11–13]. The body stimulates angiogenesis partly by producing VEGF [14, 15] as a biological reaction to hypoxia and oxidative stress. Thus, VEGF production by cells that are not infected with EB virus might mask the association between EBV EA-IgG titer and atherosclerosis among older individuals. However, genetic characteristics related to lower production of VEGF might weaken this masking effect.

The number of (T) alleles in VEGF polymorphism rs3025039 is reported to be inversely associated with serum VEGF concentration among healthy individuals [16]. Our previous study revealed that rs3025039 (T) allele carriers are less likely to develop atherosclerosis [17]. Therefore, VEGF polymorphism rs3025039 status might influence the association between EBV EA-IgG titer and atherosclerosis.

Therefore, we hypothesized that VEGF polymorphism rs3025039 status influences the association between EBV EA-IgG titer and atherosclerosis. We also hypothesized that a positive association between EBV EA-IgG titer and atherosclerosis could be observed only among rs3025039 (T) allele carriers.

Clarifying those hypotheses is an efficient tool for understanding the potential mechanism underlying the association between EB virus infection and atherosclerosis. Therefore, a cross-sectional study was conducted.

## 2. Materials and methods

### 2.1. Study population

The methods related to the present risk surveys, including genetic (polymorphism) data [17, 18] and EBV EA-IgG [19], have been described elsewhere.

The entire population of Goto City could not be surveyed in the span of 1 year because of a shortage of study staff engaged in the present survey. Therefore, to ensure that all areas of the city were included, we conducted the survey in different parts of the city over 3 years. Details about the present study have been described elsewhere [18, 19].

The study population comprised 2,828 individuals (1,066 men and 1,762 women) aged 60–89 years from Goto City in southwestern Japan who had previously undergone an annual health check-up conducted by the local government under the direction of the Ministry of Health, Labour and Welfare of Japan during 2017–2019.

By using a double-blind procedure, the targeted pollution of this study is collected.

Individuals without data on EBV EA-IgG titer (n = 33) were excluded. Individuals without data on rs3025039 genotype (n = 128) or rs3025020 genotype (n = 6) were also excluded. The remaining 2,661 elderly Japanese individuals (987 men and 1,674 women) with a mean age of 73.0 ± standard deviation (SD) 7.3 years were included in the study.

To ensure that the participants understood the study’s objective, written consent forms were used. Informed consent was obtained from all participants. All procedures performed in studies involving human participants were in accordance with the ethical standards of the institutional research committee and with the 1964 Helsinki Declaration and its later amendments or comparable ethical standards. This study was approved by the Ethics Committee for Human Use of Nagasaki University (project registration number 14051404).

### 2.2. Data collection and laboratory measurements

Information on habits was obtained from each participant by trained interviewers. Body weight and height were measured using an automatic body composition analyzer (BF-220; Tanita, Tokyo, Japan). Body mass index (BMI; kg/m^2^) was calculated. After at least 5 minutes of rest, blood pressure in the sitting position was measured using a blood pressure measuring device (HEM-907; Omron, Kyoto, Japan).

Fasting blood samples were obtained from each participant. Concentrations of triglycerides, high-density lipoprotein cholesterol (HDLc), and hemoglobin A1c (HbA1c) were measured using standard procedures at SRL, Inc. (Tokyo, Japan).

A fully automated genomic DNA isolation system (Gene Prep Star NA-480, Kurabo Industries Ltd., Osaka, Japan) was used to extract genomic DNA from 2 mL of whole peripheral blood. Genotyping to identify the single nucleotide polymorphisms rs3025039 and rs3025020 was performed using TaqMan assays and a LightCycler 480 thermal cycling platform (Roche Diagnostics, Basel, Switzerland). To detect EBV EA-IgG, an enzyme immunoassay kit (Denka Inc., Niigata, Japan) was used. This kit detects anti-EA antibodies that reflect a diffuse pattern of EA (D) IgG. Standard calibrators were used in each assay to calculate the index value/optical density ratio, which served as a semiquantitative measure of antibody levels. All assays met predetermined quality control measures based on positive, negative, and blank controls.

Experienced vascular technicians measured carotid intima-media thickness (CIMT) of the left and right common carotid arteries using ultrasonography (LOGIQ Book XP) with a 10-MHz transducer (GE Healthcare, Milwaukee, WI, USA). The maximum CIMT values for the left and right common carotid arteries were calculated using digital edge-detection software (Intimascope; MediaCross, Tokyo, Japan) [20]. Because CIMT <1.1 mm was reported as normal [21], atherosclerosis was defined as CIMT ≥1.1 mm, as in previous studies [10, 17].

### 2.3. Statistical analysis

Clinical characteristics related to EBV EA-IgG titers and rs3025039 genotypes were expressed as means ± SD for continuous variables such as age, BMI, systolic blood pressure, HDLc, and HbA1c. Since triglycerides had a skewed distribution, the median [interquartile range] was reported.

The prevalence of male gender, drinking, smoking, rs3025020 CT-heterozygote status, and rs3025020 TT-homozygote status stratified by EBV EA-IgG titer tertile and rs3025039 genotype were also calculated.

Significant differences were evaluated using analysis of variance (ANOVA) for continuous variables and the chi-squared test for proportions.

Logistic regression was used to calculate odds ratios (ORs) and 95% confidence intervals (CIs) for atherosclerosis with EBV EA-IgG titer in relation to rs3025039 genotype. Using logistic regression, the influence of rs3025039 (T) allele carrier status on the association between EBV EA-IgG titer and atherosclerosis was also evaluated.

Non-classical cardiovascular risk factors that directly influence the intravascular environment are important for the present analysis. Therefore, in addition to sex and age, smoking status, which is a classical cardiovascular risk factor that indicates carbon monoxide exposure, was included [22]. Because exposure to carbon monoxide is directly associated with CIMT [23], smoking status should act as a confounder in the present model. In addition, alcohol consumption, which is related to the serum concentration of ethanol, has also been reported to be associated with CIMT [24]. Thus, in addition to BMI and factors related to hypertension, dyslipidemia, or diabetes, smoking status and drinking status should act as confounders in the present analysis. Because variables that directly influence the intravascular environment should be treated as confounders in the present analysis, medications for hypertension, dyslipidemia, and diabetes were not included as confounders. Since values of systolic blood pressure, triglycerides, HDLc, and HbA1c could influence the use of medications, instead of adjusting for medication use, we adjusted for those factors.

**Fig. 1:**
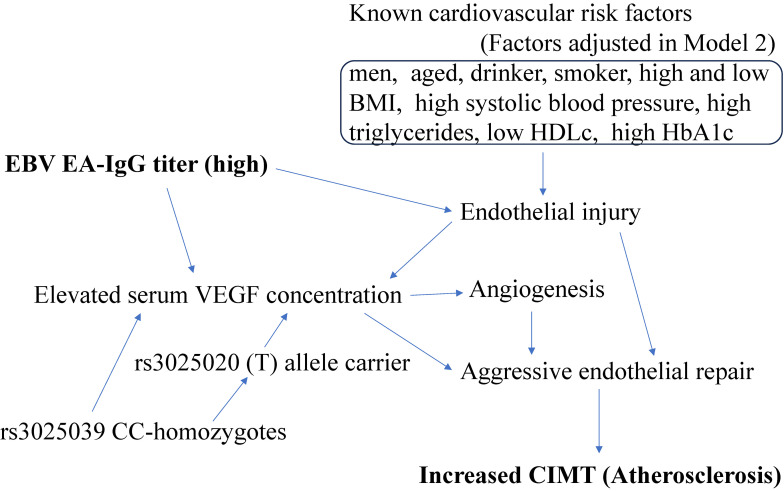
Directed acyclic graph-informed regression model (Model 3) BMI: body mass index. CIMT: carotid intima-media thickness. EBV EA-IgG: anti-early antigen EB virus IgG. VEGF: vascular endothelial growth factor. HDLc: high density lipoprotein-cholesterol.

Three different approaches were used to adjust for confounding factors. Model 1 adjusted only for sex and age. Model 2 included several other potential confounding factors in addition to sex and age, namely drinking status (non-drinker, drinker), smoking status (non-smoker, smoker), BMI (kg/m^2^), systolic blood pressure (mmHg), triglycerides (mg/dL), HDLc (mg/dL), and HbA1c (%). Model 3 further adjusted for rs3025020 genotype because the number of (T) alleles in VEGF polymorphism rs3025020 has been reported to be positively associated with serum VEGF concentration among healthy individuals [16]. Model 3 is the same as the one used in a previous study that evaluated the association between VEGF polymorphism rs3025039 and atherosclerosis among older Japanese aged 60 to 89 years [17]. Figure 1 shows a directed acyclic graph-informed regression model (Model 3).

Values of p < 0.05 were regarded as statistically significant. All statistical analyses were performed with SAS for Windows (version 9.4; SAS Inc., Cary, NC, USA).

## 3. Results

### 3.1. Characteristics of the study population

Among the study population, there were 1,731 rs3025039 CC-homozygotes, 790 CT-heterozygotes, and 140 TT-homozygotes. Characteristics of the study population by EBV EA-IgG titer tertile are shown in Table 1. No significant associations between EBV EA-IgG titer and the variables evaluated were found.

**Table 1:** Characteristics of the study population by EBV EA-IgG titer

	**Anti-early antigen IgG Epstein-Barr virus titer**			**p**

	**Low** **(T1)**	**Medium** **(T2)**	**High** **(T3)**	
Number of participants	638	1,097	926	
Men, %	37.5	34.9	39.4	0.110
Age, years	72.6 ± 7.1	73.0 ± 7.3	73.3 ± 7.1	0.220
Current smoker, %	5.6	7.8	8.0	0.160
Current drinker, %	31.0	28.4	28.5	0.462
BMI, kg/m^2^	23.0 ± 3.3	23.1 ± 3.3	23.0 ± 3.5	0.678
Systolic blood pressure, mmHg	134 ± 17	134 ± 18	135 ± 17	0.075
Triglycerides, mg/dL	87 [67 124]^*1^	88 [64 123]^*1^	89 [86 124]^*1^	0.666^*2^
HDL cholesterol	62 ± 15	62 ± 15	61 ± 16	0.409
HbA1c, %	5.8 ± 0.6	5.8 ± 0.5	5.8 ± 0.5	0.403
rs3025020 (C/T)	37.0	38.7	39.5	0.595
rs3025020 (T/T)	9.7	10.0	7.2	0.069

Unless otherwise indicated, means ± standard deviation are presented. *1: Values are medians [interquartile range]. *2: Logarithmic transformation was used for evaluating p. BMI: body mass index. HDLc: high density lipoprotein cholesterol. HbA1c: hemoglobin.

Table 2 shows the characteristics of the study participants by rs3025039 genotype. The number of (T) alleles in rs3025039 was significantly inversely associated with age, rs3025020 CT-heterozygote status, and rs3025020 TT-homozygote status.

**Table 2:** Characteristics of the study population by rs3025039 genotype

	**rs3025039**			**p**

	**C/C**	**C/T**	**T/T**	
Number of participants	1,731	790	140	
Men, %	36.9	37.2	38.6	0.923
Age, years	73.3 ± 7.2	72.4 ± 7.0	72.3 ± 7.6	0.009
Current smoker, %	7.3	7.5	7.9	0.960
Current drinker, %	29.0	29.9	25.7	0.602
BMI, kg/m^2^	23.0 ± 3.4	22.9 ± 3.3	23.1 ± 3.4	0.817
Systolic blood pressure, mmHg	135 ± 17	133 ± 18	132 ± 17	0.060
Triglycerides, mg/dL	88 [65 124]^*1^	88 [67 124]^*1^	86 [65 121]^*1^	0.586^*2^
HDL cholesterol	62 ± 15	62 ± 15	61 ± 16	0.409
HbA1c, %	5.8 ± 0.5	5.8 ± 0.5	5.7 ± 0.5	0.530
rs3025020 (C/T)	43.4	33.8	6.4	<0.001
rs3025020 (T/T)	13.6	0.5	0.0	<0.001

Unless otherwise indicated, means ± standard deviation are presented. *1: Values are medians [interquartile range]. *2: Logarithmic transformation was used for evaluating p. BMI: body mass index. HDLc: high-density lipoprotein cholesterol. HbA1c: hemoglobin A1c.

### 3.2. Association between EBV EA-IgG titer and atherosclerosis in relation to rs3025039 genotype

Table 3 shows the ORs (95% CIs) for atherosclerosis of EBV EA-IgG titer among rs3025039 CC-homozygotes. Among individuals with rs3025039 CC-homozygotes, no significant associations between EBV EA-IgG titer and atherosclerosis were observed. Table 4 shows the ORs (95% CIs) for atherosclerosis of EBV EA-IgG titer among rs3025039 CT-heterozygotes and TT-homozygotes. A significant positive association was observed for rs3025039 CT-heterozygotes. For rs3025039 TT-homozygotes, although power in Models 1 did not reach statistical significance, a positive tendency between EBV EA-IgG titer and atherosclerosis was observed. In Model 2 and 3, significant positive associations was observed.

**Table 3:** Atherosclerosis and EBV EA-IgG among rs3025039 CC-homozygotes

	**Anti-early antigen IgG Epstein-Barr virus titer**					**1 SD increment in** **EBV EA-IgG titer**		p*

	**Low (T1)**	**Medium (T2)**		**High (T3)**				
		
		**OR** **(95% CI)**	**p**	**OR** **(95% CI)**	**p**	**OR** **(95% CI)**	**p**	
**rs3025039 CC-homozygotes**								
Number of participants	423	723		585		1731		
Number of cases, %	86 (20.3)	165 (22.8)		131 (22.4)		382 (22.1)		
Model 1	Reference	1.13 (0.84, 1.54)	0.418	1.08 (0.79, 1.48)	0.628	0.97 (0.86, 1.09)	0.595	0.372
Model 2	Reference	1.11 (0.82, 1.50)	0.509	1.07 (0.78, 1.47)	0.672	0.97 (0.86, 1.10)	0.668	0.343
Model 3	Reference	1.11 (0.82, 1.50)	0.509	1.07 (0.78, 1.47)	0.673	0.97 (0.86, 1.10)	0.665	0.295

*: p value for goodness of fit calculated using the Hosmer-Lemeshow test. Model 1: adjusted only for sex and age. Model 2: further adjusted for smoking status, drinking status, body mass index, systolic blood pressure, triglycerides, high density lipoprotein (HDL) cholesterol, and HbA1c. Model 3: included variables in Model 2 and rs3025020 genotype. 1 SD increment in EBV EA-IgG titer: 1 standard deviation (SD) increment in anti-early antigen IgG Epstein-Barr virus titer, CI, confidence interval; OR: odds ratio.

**Table 4:** Atherosclerosis and EBV EA-IgG among rs3025039 CT-heterozygotes and TT-homozygotes

	**Anti-early antigen IgG Epstein-Barr virus titer**					**1 SD increment in ** **EBV** ** EA-IgG titer**		p*

	**Low (T1)**	**Medium (T2)**		**High (T3)**				
		
		**OR (95% CI)**	**p**	**OR (95% CI)**	**p**	**OR (95% CI)**	**p**	
**rs3025039 CT-heterozygotes**								
Number of participants	169	326		295		790		
Number of cases, %	25 (14.8)	57 (17.5)		68 (23.1)		150 (19.0)		
Model 1	Reference	1.34 (0.79, 2.26)	0.279	1.70 (1.01, 2.86)	0.044	1.17 (1.01, 1.35)	0.033	0.658
Model 2	Reference	1.38 (0.81, 2.36)	0.239	1.67 (0.98, 2.83)	0.058	1.18 (1.02, 1.37)	0.023	0.192
Model 3	Reference	1.37 (0.80, 2.35)	0.246	1.65 (0.97, 2.80)	0.063	1.18 (1.02, 1.37)	0.024	0.183
**rs3025039 TT-homozygotes**								
Number of participants	46	48		46		140		
Number of cases, %	4 (8.7)	7 (14.6)		12 (26.1)		23 (16.4)		
Model 1	Reference	1.67 (0.44, 6.31)	0.454	3.18 (0.91, 11.13)	0.071	1.17 (0.72, 1.91)	0.533	0.632
Model 2	Reference	1.73 (0.40, 7.50)	0.463	3.85 (1.00, 14.83)	0.050	1.23 (0.72, 2.08)	0.451	0.513
Model 3	Reference	1.65 (0.38, 7.04)	0.502	3.90 (1.01, 14.98)	0.048	1.25 (0.73, 2.15)	0.409	0.438

*: p value for goodness of fit calculated using the Hosmer-Lemeshow test. Model 1: adjusted only for sex and age. Model 2: further adjusted for smoking status, drinking status, body mass index, systolic blood pressure, triglycerides, high density lipoprotein (HDL) cholesterol, and HbA1c. Model 3: included variables in Model 2 and rs3025020 genotype. 1 SD increment in EBV EA-IgG titer: 1 standard deviation (SD) increment in anti-early antigen IgG Epstein-Barr virus titer, CI, confidence interval; OR: odds ratio.

### 3.3. Association between EBV EA-IgG titer and atherosclerosis among rs3025039 (T) allele carriers

Positive associations between EBV EA-IgG titer and atherosclerosis were observed among rs3025039 CT-heterozygotes and rs3025039 TT-homozygotes, as shown in Table 4. Therefore, among participants rs3025039 (T) allele carriers, there was a positive association between EBV EA-IgG titer and atherosclerosis.

Table 5 shows the association between EBV EA-IgG titer and atherosclerosis among rs3025039 (T) allele carriers. A significant association between EBV EA-IgG titer and atherosclerosis was observed.

**Table 5:** Atherosclerosis and EBV EA-IgG among rs3025039 (T) allele carriers

	**Anti-early antigen IgG Epstein-Barr virus titer**					p*	**1 SD increment in** **EBV EA-IgG titer**		p*

	**Low (T1)**	**Medium (T2)**		**High (T3)**					
		
		**OR (95% CI)**	**p**	**OR (95% CI)**	**p**		**OR (95% CI)**	**p**	
Number of participants	215	374		341			930		
Number of cases (%)	29 (13.5)	64 (17.1)		80 (23.5)			173 (18.6)		
Model 1	Reference	1.41 (0.86, 2.29)	0.172	1.88 (1.17, 3.04)	0.009	0.192	1.17 (1.02, 1.34)	0.023	0.270
Model 2	Reference	1.45 (0.88, 2.37)	0.144	1.89 (1.17, 3.07)	0.010	0.390	1.18 (1.03, 1.36)	0.017	0.408
Model 3	Reference	1.44 (0.88, 2.36)	0.153	1.88 (1.15, 3.05)	0.011	0.577	1.18 (1.03, 1.36)	0.018	0.168

*: p value for goodness of fit calculated using the Hosmer-Lemeshow test. Model 1: adjusted only for sex and age. Model 2: further adjusted for smoking status, drinking status, body mass index, systolic blood pressure, triglycerides, high density lipoprotein (HDL) cholesterol, and HbA1c. Model 3: included variables in Model 2 and rs3025020 genotype. 1 SD increment of EBV EA-IgG titer: 1 standard deviation (SD) increment in anti-early antigen IgG Epstein-Barr virus titer, CI, confidence interval; OR: odds ratio.

Among rs3025039 (T) allele carriers, with the lowest tertile (T1) of EBV EA-IgG titer as the reference group, the age- and sex-adjusted (Model 1) had an OR (95% CI) for atherosclerosis of 1.41 (0.86, 2.29) for the medium tertile (T2); it was 1.88 (1.17, 3,04) for the highest tertile (T3). These associations remained even after adjustment for known potential confounders. In the fully adjusted (Model 3), the OR (95% CI) was 1.44 (0.88, 2.36) for T2 and 1.88 (1.15, 3.05) for T3.

### 3.4. Effect of rs3025039 (T) allele carrier status on the association between EBV EA-IgG titer and atherosclerosis

We also found that rs3025039 (T) allele carrier status affected the association between EBV EA-IgG titer and atherosclerosis. The adjusted p value for the effect of this interaction on the association between EBV EA-IgG titer tertile and atherosclerosis was 0.046 for Model 1, 0.048 for Model 2, and 0.0496 for Model 3. The corresponding values between a 1 SD increment in EBV EA-IgG titer and atherosclerosis were 0.043, 0.048, and 0.0497, respectively.

## 4. Discussion

The major finding of the present study is that EBV EA-IgG titer is significantly positively associated with atherosclerosis based on CIMT only among participants who are genetically less likely to have progressive angiogenesis. The association between EBV EA-IgG titer and atherosclerosis was significantly affected by rs3025039 (T) allele carrier status.

This is the first study that revealed an association between EBV EA-IgG titer and atherosclerosis as evaluated with CIMT. In this study, being genetically less likely to have progressive angiogenesis was revealed to be a determinant of the association between EB virus titer and atherosclerosis.

Endothelial cells are important targets for EB virus infection [25]. Upregulated expression of vascular cell adhesion molecule-1 (VCAM-1) on cytokine-stimulated endothelial cells might initiate vascular dysfunction [26], partly by inducing adhesion of EB virus-positive natural killer cells [27]. EBV EA-IgG titer indicates the presence of a chronic immunological response. Therefore, EBV EA-IgG titer might indicate the magnitude of endothelial cell injury. Since increased CIMT reflects the process of endothelial repair [28], EBV EA-IgG titer could be positively associated with atherosclerosis as evaluated with CIMT. However, the mechanism by which EBV EA-IgG titer indicates the magnitude of endothelial cell injury might not be the main explanation for the association between EBV EA-IgG titer and atherosclerosis because no significant associations were observed among VEGF polymorphism rs3025039 CC-homozygotes.

**Fig. 2:**
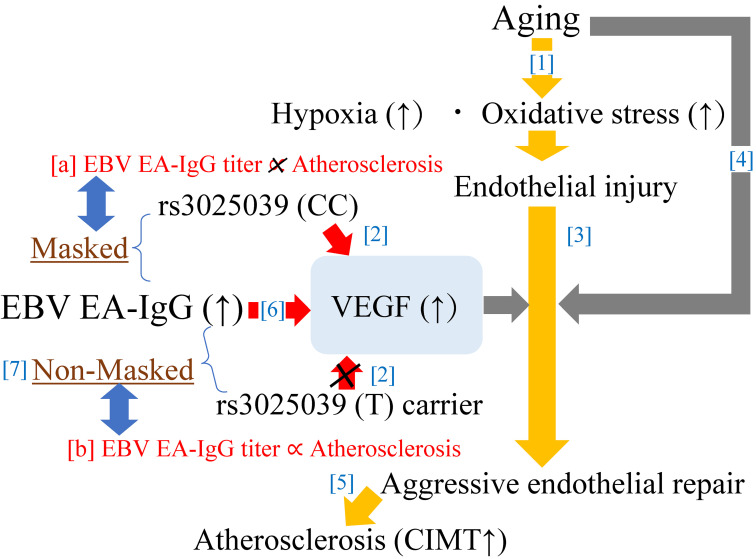
Potential mechanisms underlying the EBV EA-IgG and atherosclerosis The relationships marked in red [a] and [b] were observed in this study. Relationships marked in blue [1 to 7] were reported in previous studies. CIMT: carotid intima-media thickness. EB: Epstein-Barr. EBV EA-IgG: anti-early antigen EB virus IgG. VEGF: vascular endothelial growth factor.

Figure 2 shows the potential mechanism underlying the present results. Genetically higher producibility of VEGF might mask the association between EBV EA-IgG titer and atherosclerosis.

The number of (T) alleles in VEGF polymorphism rs3025039 is reported to be inversely associated with serum VEGF concentration among healthy individuals [16] (Fig. 2-[2]). VEGF plays a central role in progressive angiogenesis [8]. Since vasa vasorum angiogenesis is necessary for the development of atherosclerosis [9], rs3025039 (T) allele carriers are genetically less likely to have progression of atherosclerosis [17].

Aging is a process that increases hypoxia and oxidative stress [11–13] (Fig. 2-[1]), increasing the need for endothelial repair (Fig. 2-[3]). However, aging also decreases endothelial repair activity [10] (Fig. 2-[4]). Because progression of atherosclerosis involves aggressive endothelial repair [28] (Fig. 2-[5]), older individuals who are genetically less likely to have development of atherosclerosis might also have a disadvantage in maintaining endothelial function.

An elevated EBV EA-IgG titer indicates active infection. Cells infected with EB virus stimulate angiogenesis [29] by producing VEGF [30] (Fig. 2-[6]). Therefore, among participants with lower VEGF production due to genetic factors, the activity of cells infected with EB virus might determine the serum concentration of VEGF (Fig. 2-[7]), which contributes to the progression of atherosclerosis. Therefore, EBV EA-IgG titer was only associated with atherosclerosis among those with a lower capacity for producing VEGF related to VEGF polymorphism rs3025039 (T) allele status (Fig. 2-[a][b]).

In present study, for rs3025039 CT-heterozygotes, EBV EA-IgG titer as a continuous variable was revealed to be significantly positively associated with atherosclerosis. Among rs3025039 TT-homozygotes, although the analysis was underpowered, a positive tendency between EBV EA-IgG titer as a continuous variable and atherosclerosis was observed. Since essentially the same associations were observed among rs3025039 CT-heterozygotes and among rs3025039 TT-homozygotes, they were grouped together based on VEGF polymorphism rs3025039 (T) allele status to evaluate the relationship between EBV EA-IgG titer and atherosclerosis.

This is the first study that clarifies the influence of an angiogenesis-related genetic factor on the association between viral infection and atherosclerosis. This novel knowledge can clarify the mechanism underlying the development of atherosclerosis among EB virus carriers and introduce a novel concept, namely that the capacity for progressive angiogenesis is a key factor that determines the association between viral infection and atherosclerosis.

Most people in Japan are infected with EB virus by the time they reached adulthood and latent infection persists throughout their lifetime [31]. The target population of this study is older people aged 60–89 years. The present study aimed to evaluate the influence of a genetic factor on the association between EB virus titer and atherosclerosis as defined by CIMT. Therefore, although the present study is a cross-sectional study, it established that rs3025039 (T) allele carrier status has a significant effect on the interaction between EBV EA-IgG titer and atherosclerosis. Therefore, the fact that a VEGF-related genetic factor determines the association between EBV EA-IgG titer and atherosclerosis suggests that VEGF activity might mediate the present associations. Therefore, VEGF is not a factor that confounds the present associations.

Although serum VEGF concentration might affect the present associations, unlike genetic factors, serum VEGF concentration could influence the current status of older individuals. Angiogenesis is necessary for the development of atherosclerotic lesions [9, 10]. However, previous our study with Japanese men aged 60 to 69 years revealed significant inverse association between baseline atherosclerosis and active arterial wall thickening defined as increased values of CIMT ≥0.01 mm/year [32]. This previous study also found that due to the consumption of endothelial progenitor cells, elderly individuals with atherosclerosis at baseline could not progress CIMT anymore [32]. Endothelial progenitor cells contribute to angiogenesis [33]. A strong association between VEGF and endothelial progenitor cells has been reported [34]. Therefore, serum VEGF concentration could be strongly influenced by the current condition of elderly individuals while EB virus infection and genetic factors could influence the endothelium over a long period of time with progression to atherosclerosis. Thus, although VEGF might affect the main results of the present study, measuring serum VEGF concentration at one point in time might be inappropriate for evaluating how activity of angiogenesis influences the association between EB virus titer and atherosclerosis in relation to VEGF polymorphisms among older individuals.

Participants of this study were members of the general population in Japan, where chronic active EB virus infection is rare [35]. Therefore, most participants in this study did not chronic active EB virus infection. However, in the present study, EBV EA-IgG titer was revealed to influence the endothelium, possibly by enhancing angiogenesis. This indicates that even among asymptomatic individuals in the general population, high EBV EA-IgG titers might have a physiological effect on the endothelium. Thus, participants who have stable metabolic factors but increased CIMT should have their EBV EA-IgG titers checked when there is a suspicion that a chronic immunological response to EB virus is occurring.

Local characteristics of the study population should also be taken into consideration. Older individuals with the rs3025039 (T) allele were reported to be inversely associated with HTLV-1 infection [36], and the prevalence of HTLV-1 carriers was higher in southwestern Japan [37]. VEGF polymorphism rs3025020 was associated with a feeling of incomplete bladder emptying among older community-dwelling individuals [38]. Furthermore, VEGF polymorphism rs3025020 was also revealed to be associated with short stature and hypertension [39]. Hypertension, angiogenesis, and short stature are also associated with atherosclerosis [40, 41]. Not only the genetic factor but also the environment during childhood, including financial status and educational status, should influence the height level, which is closely associated with endothelial health, such as atherosclerosis [18]. There are many tobacco farmers in Goto City. Smoking also induces height loss [42], while endothelial maintenance capacity, including angiogenesis and atherosclerosis, is associated with height loss [43, 44]. Therefore, those factors also could influence the present results. To clarify the influence of local differences on the association between EBV EA-IgG titer and atherosclerosis in relation to rs3025039 genotype, further area-specific investigations with those data are necessary.

The clinical implication of the present study is that angiogenesis-related genetic factors influence the association between EB virus titer and atherosclerosis, as evaluated by CIMT. Therefore, the physical capacity of angiogenesis should play a significant role in the development of CIMT.

A potential limitation of the present study is that because of the small number of rs3025039 TT-homozygotes (n = 140), a statistically meaningful analysis of EBV EA-IgG titer and atherosclerosis could not be performed among those participants. However, even the analysis was underpowered, essentially same the association was observed among rs3025039 CT-heterozygotes. To clarify the differences between rs3025039 CT-heterozygotes and rs3025039 TT-homozygotes, further investigation with more participants is necessary. Because of the cross-sectional nature of this study, a causal relationship between EBV EA-IgG titer and atherosclerosis could not be established. However, an angiogenesis-related genetic factor can have a causal effect on the association between EBV EA-IgG titer and atherosclerosis. Therefore, the capacity to have progressive angiogenesis might be underlying the association between EBV EA-IgG titer and atherosclerosis.

## 5. Conclusion

EBV EA-IgG titer was significantly positively associated with atherosclerosis as evaluated with CIMT only among participants who are genetically less likely to have progressive angiogenesis. An angiogenesis-related genetical factor was revealed to be a determinant of the association between EB virus titer and atherosclerosis. These findings introduce a novel concept that could explain the association between viral infection and atherosclerosis.

## Abbreviations

EB: Epstein-Barr
VEGF: vascular endothelial growth factor
EBV EA-IgG: anti-early antigen EB virus IgG
SD: standard deviation
BMI: body mass index
HDLc: high-density lipoprotein cholesterol
HbA1c: hemoglobin A1c
CIMT: carotid intima-media thickness
ANOVA: analysis of variance
OR: odds ratio
Cis: confidence interval
T1: lowest tertile
T2: medium tertile
T3: high tertile
VCAM-1: vascular cell adhesion molecule-1

## Declarations

### Availability of data and material

For our studies described in this review, we cannot publicly provide individual data due to participant confidentiality considerations, according to ethical guidelines in Japan. Additionally, obtaining informed consent does not include a provision for publicity sharing data. Qualified researchers may apply to access a minimal dataset. Please contact the office of data management at ritouken@vc.fctv-net.jp. Information for the data request is also available at http://www.med.nagasaki-u.ac.jp/cm/.
